# A Role for Oxidized DNA Precursors in Huntington's Disease–Like Striatal Neurodegeneration

**DOI:** 10.1371/journal.pgen.1000266

**Published:** 2008-11-21

**Authors:** Gabriele De Luca, Maria Teresa Russo, Paolo Degan, Cecilia Tiveron, Andrea Zijno, Ettore Meccia, Ilenia Ventura, Elisabetta Mattei, Yusaku Nakabeppu, Marco Crescenzi, Rita Pepponi, Antonella Pèzzola, Patrizia Popoli, Margherita Bignami

**Affiliations:** 1 Department of Environment and Primary Prevention, Experimental Carcinogenesis Division, Istituto Superiore di Sanità, Rome, Italy; 2 Department of Translational Oncology, Istituto Nazionale per la Ricerca sul Cancro (IST-CBA), Genova, Italy; 3 European Brain Research Institute, Rome, Italy; 4 Institute of Neurobiology and Molecular Medicine, CNR, Rome, Italy; 5 Division of Neurofunctional Genomics, Medical Institute of Bioregulation, Kyushu University, Fukuoka, Japan; 6 Department of Drug Research and Evaluation, Central Nervous System Pharmacology Division, Istituto Superiore di Sanità, Rome, Italy; University of Minnesota, United States of America

## Abstract

Several human neurodegenerative disorders are characterized by the accumulation of 8-oxo-7,8-dihydroguanine (8-oxodG) in the DNA of affected neurons. This can occur either through direct oxidation of DNA guanine or via incorporation of the oxidized nucleotide during replication. Hydrolases that degrade oxidized purine nucleoside triphosphates normally minimize this incorporation. hMTH1 is the major human hydrolase. It degrades both 8-oxodGTP and 8-oxoGTP to the corresponding monophosphates. To investigate whether the incorporation of oxidized nucleic acid precursors contributes to neurodegeneration, we constructed a transgenic mouse in which the human hMTH1 8-oxodGTPase is expressed. hMTH1 expression protected embryonic fibroblasts and mouse tissues against the effects of oxidants. Wild-type mice exposed to 3-nitropropionic acid develop neuropathological and behavioural symptoms that resemble those of Huntington's disease. hMTH1 transgene expression conferred a dramatic protection against these Huntington's disease–like symptoms, including weight loss, dystonia and gait abnormalities, striatal degeneration, and death. In a complementary approach, an in vitro genetic model for Huntington's disease was also used. hMTH1 expression protected progenitor striatal cells containing an expanded CAG repeat of the *huntingtin* gene from toxicity associated with expression of the mutant *huntingtin*. The findings implicate oxidized nucleic acid precursors in the neuropathological features of Huntington's disease and identify the utilization of oxidized nucleoside triphosphates by striatal cells as a significant contributor to the pathogenesis of this disorder.

## Author Summary

The oxidized purine 8-oxo-7,8-dihydroguanine (8-oxodG) is often found at high levels in DNA of post-mortem brains of patients afflicted by Huntington's disease (HD) and other neurodegenerative conditions. It has been unclear, however, whether this damage accumulation is a consequence or a cause of neurodegeneration. We have addressed this problem in a transgenic mouse model in which the human hMTH1 8-oxodGTPase, which hydrolyzes oxidized nucleoside triphosphates to prevent the incorporation of oxidized bases into nucleic acids, was expressed in several organs. We report that high-level hMTH1 expression in the transgenic mouse brain provides robust protection against neurodegeneration as well as HD-like behavioural and neuropathological symptoms in a chemical model of HD (the mitochondrial toxin 3-nitropropionic acid). In a complementary approach, we used a genetic model for HD based on the use of progenitor striatal cells containing an expanded *CAG* repeat of the *huntingtin* gene. Also, in this case, hMTH1 provided strong protection against cell death associated with expression of the mutant huntingtin protein. The new findings we report shed important light on the connection between oxidative DNA damage and human neurodegenerative disorders. Our findings go some way towards forging a causative link between neurodegeneration and oxidative DNA/RNA damage and identify the oxidized purine nucleoside triphosphates as important contributors.

## Introduction

Mammalian cells assign considerable resources to protecting their DNA against mutagenic oxidative damage by the reactive oxygen species (ROS) that are an inevitable by-product of oxidative metabolism. An imbalance in the production and detoxification of ROS can lead to a condition of oxidative stress and the accumulation of DNA lesions. DNA 8-oxo-7,8-dihydroguanine (8-oxodG) is a marker of DNA oxidation and shares with other oxidized bases the ability to miscode during replication to generate base substitution mutations. The accumulation of DNA 8-oxodG is associated with genome instability and increased cancer incidence [1],[2].

Endogenous DNA damage caused by ROS is also considered to be important in the etiology of several neurodegenerative disorders. Recent evidence indicates that defects in the repair of DNA damage produced by oxidative stress can lead to neuronal cell death. Thus, rare neurodegenerative diseases such as ataxia with oculomotor apraxia 1 (AOA1) or spinocerebellar ataxia with axonal neuropathy 1 (SCAN1) are defective in genes (*aprataxin* (*APTX*) and tyrosyl-DNA phosphodiesterase 1 (*TDP1*), respectively) encoding enzymes involved in the repair of specific types of DNA single strand breaks. It has been suggested that the accumulation of oxidative stress-induced oxidative DNA damage underlie the neurodegeneration in AOA1 and SCAN1 tissues [for review, see [3]. In addition to these genetic diseases with DNA repair defects, accumulation of oxidative damage in brain DNA is often associated with other neurodegenerative diseases such as Huntington's disease (HD) [4]–[6], Parkinson's disease (PD) [7] or amyotrophic lateral sclerosis (ALS) [8]–[9].

What is the source of this oxidative DNA damage? DNA 8-oxodG can derive from direct oxidation of DNA guanine *in situ* or *via* incorporation from the oxidized dNTP pool during replication. Mammalian cells have multiple repair mechanisms to protect their genome against the accumulation of DNA 8-oxodG. The major participants in this are the specific DNA glycosylases (OGG1, MYH and NEILs) belonging to the base excision repair (BER) pathway [10]. The nucleotide excision repair CSA and CSB proteins also contribute [11]–[13].

The second layer of protection occurs at the level of nucleic acid precursors. A family of hydrolases (NUDIX) eliminates oxidized precursors from the dNTP pool, thereby excluding them from DNA [14]. hMTH1 (human *Escherichia coli MutT* homolog) is the major human enzyme. It hydrolyzes three oxidized purine deoxynucleoside triphosphates (8-oxodGTP, 2-OH-dATP and 8-OH-dATP) to their corresponding monophosphates [15]–[16]. It can also hydrolyze the ribo forms - 8-oxoGTP and 2-OH-ATP, thereby preventing their incorporation into RNA [17],[14]. The phenotype of knockout mice confirms the importance of this protection. Homozygous inactivation of the murine *Mth1* gene leads to elevated levels of DNA 8-oxodG and an increased incidence of stomach, liver and lung cancer [18]. In addition, inactivation of murine *Mth1^−/−^* in a PD model was associated with the accumulation of excess oxidative DNA damage in dopaminergic neurons following exposure to a selective neurotoxin [19].

To investigate this phenomenon in more detail, we took the reverse approach and constructed transgenic mice expressing a high level of the human MTH1 protein. In particular, we investigated whether transgenic hMTH1 expression provided protection against another neurodegenerative disorder, HD. HD is a dominantly inherited disorder in which expansion of a CAG repeat tract that lengthens a polyglutamine segment in the coding region of the *huntingtin (htt)* gene to 37 or more residues, leads to the progressive loss of neurons in the striatum [20]. Two complementary approaches were employed. In the first, we used an experimental model for HD in which 3-nitropropionic acid (3-NP), an inhibitor of mitochondrial oxidative metabolism induces oxidative stress and causes striatal degeneration and behavioural deficits similar to those of HD [21]. In the second approach, we examined the effect of hMTH1 in a genetic model of HD, in which progenitor striatal cells from mutant knock-in mice express the expanded CAG repeats in the *htt* gene [22]. Our findings implicate oxidized nucleic acid precursors of striatal cells in the neuropathological features of HD and identify them as significant contributors to the development of this disease.

## Results

### Construction of a Transgenic Mouse Expressing the *hMTH1* cDNA

**Figure 1:**
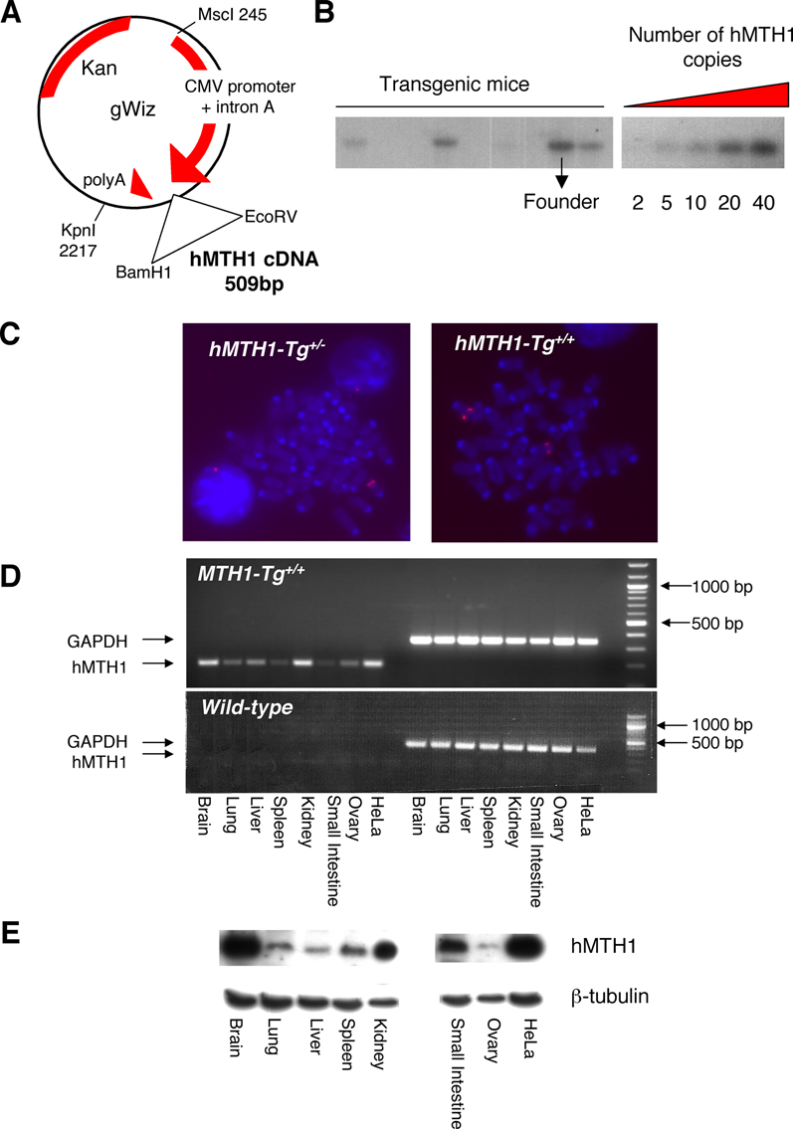
Construction and characterization of a transgenic mouse expressing the *hMTH1* cDNA. (A) A BamH1-EcoRV fragment (509 bp) derived from pcDEBΔ [23] encoding the *hMTH1* cDNA was subcloned into the gWIZ vector under the control of the CMV promoter. This vector transfected into wild-type MEFs expressed the hMTH1 protein (data not shown). The MscI-KpnI fragment (2481 bp) was used in the construction of the transgenic mouse. (B) Determination of transgene copy number. Genomic DNA from mouse tails was analysed by Southern blot analysis (left panel). In the right panel 2, 5, 10, 20, 40 copies of the MscI-KpnI fragment were analysed. Both blots were probed with the same probe. Copy number was determined by comparison. The arrow indicates the DNA from the mouse that was used as a founder for the colony. (C) Analysis by FISH of the number of hMTH1 integrations in hemizygous (*hMTH1-Tg^+/−^*) and homozygous (*hMTH1-Tg^+/+^*) strains. (D) Expression of *hMTH1* mRNA. RT-PCR was performed using total RNA from the indicated organs and human specific primers. RT-PCR for the *GADPH* gene is used as an internal control. *hMTH1* and *GAPDH* fragments are respectively 200 bp and 330 bp. (E) Western blot analysis of transgene expression. Total proteins (20–40 µg) from a range of tissues were separated by SDS polyacrylamide electrophoresis, blotted and probed with an antibody against hMTH1. β-tubulin was used as a loading control.

A 509 bp hMTH1 cDNA (BamH1-EcoRV fragment) [23] was cloned into the gWIZ vector under the control of the CMV promoter (Figure 1A) and an MscI-KpnI fragment microinjected into pronuclei of zygotes. The presence of the transgene was verified by Southern blotting of tail DNA from several founder mice. This revealed a single integration site containing between 20 and 40 copies of the transgene (Figure 1B). One of the founder mice expressing 40 copies of the hMTH1 transgene was selected (Figure 1B, see arrow) and either maintained as hemizygous (*hMTH1-Tg^+/−^*) or bred to homozygousity (*hMTH1-Tg^+/+^*). The number of hMTH1 sites confirmed by FISH (Figure 1C). Crosses between hMTH1 hemizygous produced offspring in the ratios consistent with Mendelian segregation: 7/37 (18.9%) hMTH1-Tg^+/+^, 21/37 (56.7%) hMTH1-Tg^+/−^ and 9/37 (24.3%) hMTH1-Tg^−/−^), (χ^2^ test; p≤0,05).

**Figure 2:**
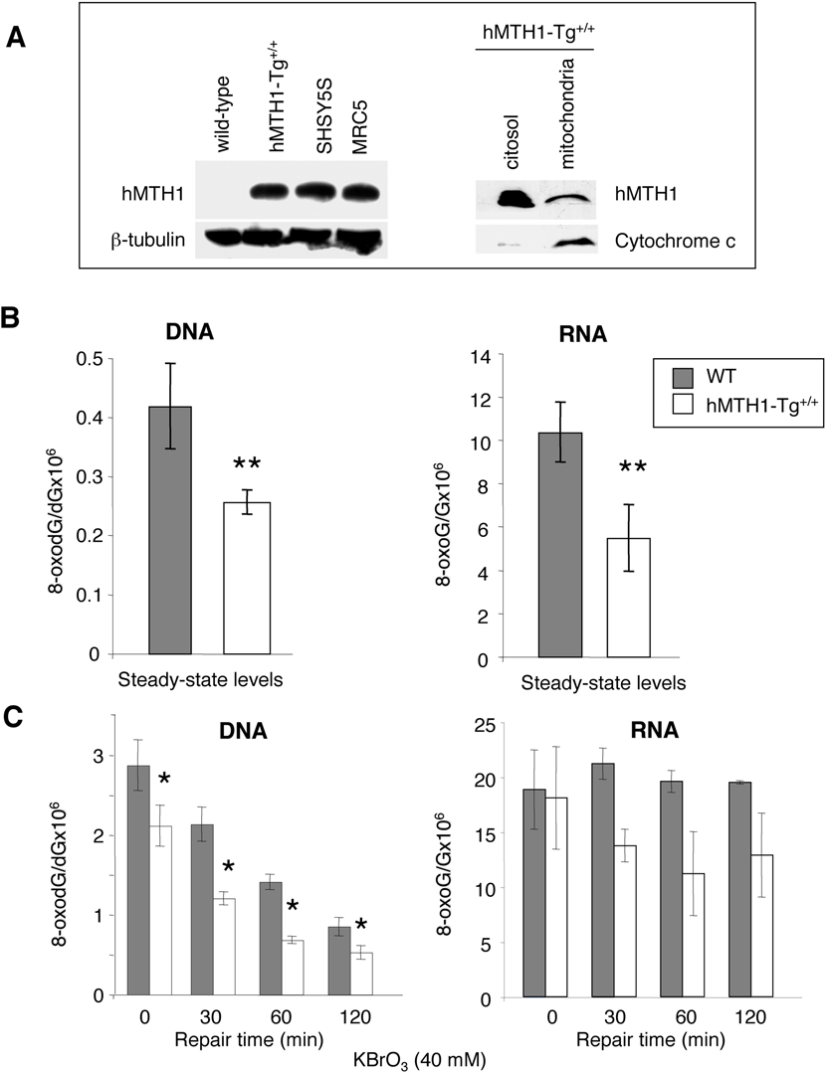
hMTH1 protein in *hMTH1-Tg^+/+^* MEFs and protection against oxidative stress. (A) Expression of hMTH1 in wild-type and homozygous *hMTH1-Tg^+/+^* MEFs. Total cell extracts were separated by 12%-SDS polyacrylamide electrophoresis, blotted and probed with an antibody against hMTH1 [23]. The human SHSY5S neuroblastoma and SV40-transformed MRC5 cell lines are shown for comparison (left panel). Western blotting of subcellular fractions of *hMTH1-Tg^+/+^* MEFs with anti-hMTH1 (right panel). Proteins were separated on 18%-SDS polyacrylamide electrophoresis and cytocrome *c* was used to quantify mitocondrial cell extracts. (B) Steady-state levels of oxidized guanine. DNA and RNA from wild-type (filled bars) and *hMTH1-Tg^+/+^* (open bars) MEFs were digested to nucleosides and 8-oxodG and 8-oxoG were separated and quantified by HPLC-EC. Values are expressed as ratio to DNA and RNA guanine, respectively. Values are the mean±SE of 3 independent determinations. Asterisks indicate statistically significant differences (p-value = 0.005 and 0.015 for DNA and RNA, respectively; *t*-test). (C) Levels of oxidized guanine following oxidant treatment. 8-oxodG and 8-oxoG were measured by HPLC-EC in DNA and RNA extracted from MEFs exposed to 40 mM KBrO_3_ for 30 min (time 0) and after the indicated times of repair incubation in drug-free medium (30, 60, 120 min). Wild type (filled bars) and *hMTH1-Tg^+/+^* (open bars). Values are the mean±SE of 3 independent determinations. Asterisks indicate a p-value≤0.05; *t*-test.

We next examined hMTH1 expression in the animal tissues. Transgene expression in several organs was first verified by RT-PCR using human-specific primers (Figure 1D). Several organs (brain, lung, liver, spleen, kidney, small intestine, ovary) expressed hMTH1 at significant levels and western blotting of tissue extracts confirmed the presence of the enzyme. No signal for the endogenous mMTH1 protein was detected using the anti-human hMTH1 antibody (data not shown and Figure 2A). hMTH1 was particularly highly expressed in brain and kidney (Figure 1E).

### hMTH1 Expression Protects MEFs against Oxidative Damage In Vitro

Embryonic fibroblasts (MEFs) were prepared from *hMTH1-Tg^+/+^* and wild-type animals and hMTH1 expression confirmed by western blotting. When the total homogenate was immunoblotted with anti-hMTH1, the antibody reacted with a single 18 KDa protein of the expected size (Figure 2A, left panel). The endogenous mouse MTH1 (mMTH1) was not detected by this antibody raised against the human protein, possibly because of its lower affinity for the mouse homolog [24]. We note that the levels of hMTH1 protein in extracts of *hMTH1-Tg^+/+^* MEFs were similar to those observed in some transformed human cell lines (SV40-transformed MRC5 cells and SHSY5S neuroblastoma cell line). It has been previously shown that the sequence encoded by the *hMTH1* cDNA has full information for the localization of the protein in mitochondria as well as in the cytoplasm [23]. To determine the intracellular localization of hMTH1 in *hMTH1-Tg^+/+^* MEFs, cells were separated into cytosolic and mitochondrial fractions and each subcellular fraction was analyzed by immunoblotting using the anti-hMTH1. Similarly to previous observations in HeLa cells [23], hMTH1 was present in both fractions indicating that the protein localizes also in mitochondria (Figure 2A, right panel).

The steady-state levels of DNA 8-oxodG in wild-type and transgenic MEFs were then examined. Transgene expression reduced DNA 8-oxodG content 1.7 fold, from 0.42±0.07 lesions per 10^6^ dG in wild-type cells, to 0.25±0.02 in *hMTH1-Tg^+/+^* MEFs (p-value = 0.005, *t*-test)(Figure 2B, left panel). hMTH1 also hydrolyzes oxidized ribonucleoside triphosphates to prevent their utilization during RNA synthesis [14]. To examine whether hMTH1 expression affected endogenous levels of RNA oxidation, total RNA was extracted from wild-type and *hMTH1-Tg^+/+^* MEFs using a protocol which allowed the simultaneous determination of both the ribo- and deoxyribonucleosides of 8-oxoG by HPLC/EC [25]. In wild-type MEFs steady-state levels of RNA 8-oxoG were 25-fold higher than DNA 8-oxodG (the values were 10.3 8-oxoG per 10^6^ G in RNA *versus* 0.4 per 10^6^ dG in DNA) (Figure 2B, right panel) [26]. hMTH1 expression in untreated transgenic MEFs was associated with a 2-fold decrease of RNA 8-oxoG compared with wild-type cells (from 10.3 to 5.5 adducts per 10^6^ G; p = 0.015, *t*-test).

This protection was also evident following exposure to an exogenous oxidant. We chose KBrO_3_ since this chemical induces very high levels of base damage [27] in comparison to other agents which induce a more general type of oxidative stress, including single and double strand breaks. In wild-type cells exposure for 30 min to 40 mM KBrO_3_ introduced into DNA 2.9±0.32 lesions per 10^6^ dG and this level of oxidation decreased over time with an half life around 60 min (Figure 2C, left panel). In *hMTH1-Tg^+/+^* cells, initial DNA 8-oxodG levels were lower and the differences were maintained during subsequent incubation (p-value<0.05, t-test). We conclude that hMTH1 expression significantly reduces the level of DNA 8-oxodG generated both endogenously and following exposure to an exogenous oxidant.

KBrO_3_ treatment increased also RNA oxidation in wild-type cells (Figure 2C, right panel). While 8-oxodG was efficiently removed from DNA, levels of RNA 8-oxoG remained unchanged at least for the initial 2 hrs post-treatment time. In contrast a general trend towards decreased levels of oxidized RNA was observed in hMTH1-expressing cells (Figure 2C). We conclude that hMTH1 expression in *hMTH1-Tg^+/+^* MEFs protects against endogenous and exogenous oxidation in both DNA and RNA.

**Figure 3:**
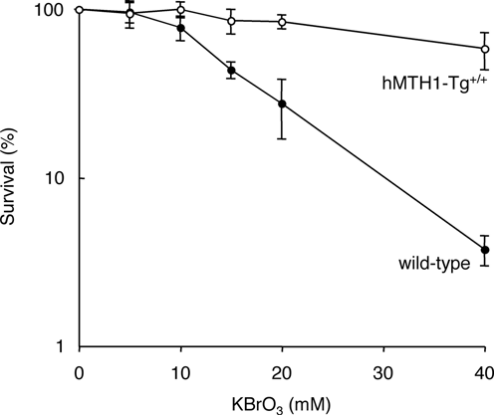
KBrO_3_ sensitivity of *hMTH1-Tg^+/+^* and wild-type MEFs. Wild-type (closed symbols) and *hMTH1-Tg^+/+^* (open symbols) MEFs were treated with KBrO_3_ for 30 min at the indicated concentrations. Viability was measured by MTT assay 48 hr later. The graphs are the mean±SD of 3 independent experiments.

hMTH1 also protected cells against DNA damage and killing by an exogenous oxidant. Cytotoxicity assays indicated that *hMTH1-Tg^+/+^* MEFs were strikingly more resistant than wild-type MEFs to killing by a range of KBrO_3_ concentrations (Figure 3). These data indicate that hMTH1 is a powerful barrier against cell death induced by an oxidative stress.

### Protective Effect of hMTH1 Expression against In Vivo Oxidative Stress

**Figure 4:**
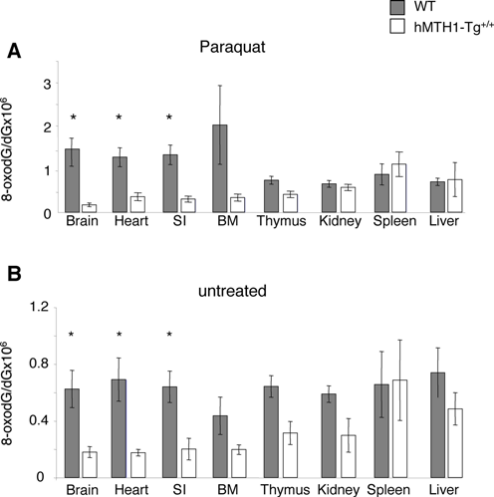
Paraquat-induced DNA 8-oxodG in wild-type and *hMTH1-Tg^+/+^* mice. (A) Groups of mice (n = 13) were injected 5 times every other day with 10 mg/kg paraquat per injection. DNA from the indicated organs was digested and levels of 8-oxodG were determined by HPLC-EC. Wild-type (filled bars); *hMTH1-Tg^+/+^* (open bars). Significant differences (Student's *t*-test) between wild-type and *hMTH1-Tg^+/+^* organs are shown with an asterisk. The *p* values were 0.01, 0.03 and 0.04 for brain, heart and small intestine, respectively. SI, small intestine. BM, bone marrow. Values are the mean±standard errors. (B) Groups of control mice (n = 10) that had received saline injections on the same schedule of Paraquat-treated animals were sacrificed 2 days after the final injection. DNA 8-oxodG was determined in various organs as described above. Wild-type (filled bars) and *hMTH1-Tg^+/+^* (open bars). The *p* values (Student's *t*-test) were 0.02, 0.01 and 0.01 for brain, heart and small intestine, respectively. Values are the mean±standard errors.

To investigate whether hMTH1 also conferred protection against oxidative DNA damage in the intact animal, oxidative stress was induced in wild-type and transgenic mice by treatment with paraquat. This pesticide is considered a model for oxidant-induced toxicity by stimulating formation of superoxide radicals [28]–[29]. Thus, paraquat-treated rats accumulate oxidative DNA damage in several tissues, including brain, lung, heart, liver and kidney [30]. Animals were treated i.p with multiple 10 mg/kg paraquat doses (a total of 5 injections every other day over 10 days). Although no specific behavioural evaluations were performed mice were indistinguishable from controls displaying no overt signs of toxicity or gross behavioural alterations. Paraquat produced high levels of DNA 8-oxodG in brain, heart, small intestine and bone marrow of wild–type animals (Figure 4A and 4B). Expression of hMTH1 in the transgenic mice conferred a significant protection against paraquat-induced oxidative DNA damage and DNA 8-oxodG levels in these organs were between 4-fold (small intestine and heart) and 9-fold (brain) lower in *hMTH1-Tg^+/+^* than in wild-type animals (p = 0.04, p = 0.03 and p = 0.01, respectively, Student's *t*-test) (Figure 4A).

The transgene also provided protection against endogenous oxidation and steady-state levels of DNA 8-oxodG in brain, heart and small intestine of untreated *hMTH1-Tg^+/+^* mice were 3.4-, 3.9-, and 3.2- fold lower than in the same tissues of wild-type animals (p values were 0.02, 0.01 and 0.01, respectively, Student's *t*-test) (Figure 4B). Thus reduced levels of 8-oxodG pools are likely to be associated with a diminished 8-oxoG incorporation during repair synthesis of endogenously incurred damage.

These findings indicate that mMTH1 activity is normally limiting in several mouse tissues, including brain. The protection conferred by hMTH1 indicates further that oxidized deoxynucleoside triphosphates are an important source of endogenous oxidative DNA damage.

### Protection by hMTH1 against HD-Like Neurodegeneration Induced by 3-NP

To examine the effect of hMTH1 expression on HD-like neurodegeneration, age-matched (8–10 weeks) transgenic and wild-type animals were treated with the mitochondrial toxin 3-NP (60 mg/kg i.p. twice a day for 5 days). This inhibitor of succinate dehydrogenase selectively causes the death of striatal neurons and induces symptoms similar to HD [21]. These include progressive weight loss, neurological abnormalities such as foot and limb dystonia, and, ultimately death.

**Figure 5:**
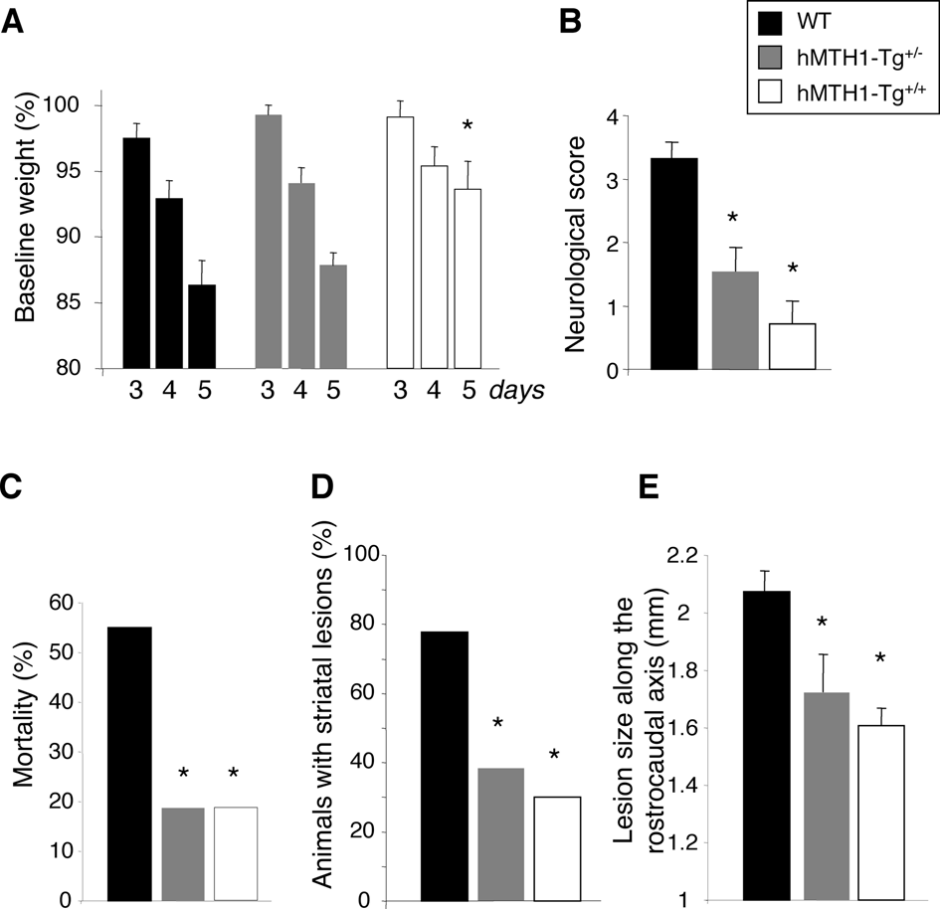
3-NP-induced toxicity in wild-type and hMTH1-Tg^+/−^ and hMTH1-Tg^+/+^ transgenic mice. Groups of wild-type (n = 20), *hMTH1-Tg^+/−^* (n = 16) and *hMTH1-Tg^+/+^* (n = 16) mice were injected i.p. twice daily for 5 days with 60 mg/kg 3-NP. Wild-type (black bars), *hMTH1-Tg^+/−^* (grey bars) and *hMTH1-Tg^+/+^* (white bars). (A) Weight loss. Body weight, measured immediately before the first injection on the indicated days, is expressed as a percentage of the pretreated body weight. (B) Motor impairment. Mice were monitored twice a day for dystonia and/or gait abnormalities. Neurological score was as follows: intermittent dystonia of one hindlimb: 1; intermittent dystonia of two hindlimbs: 2; permanent dystonia of hindimbs: 3; uncoordinated and wobbling gait or recumbency: 3; near death recumbency: 4. For each animal, the highest neurological score reached at any time of the observation period was considered. Values are mean±standard errors. (C) Cumulative mortality. The non-surviving fraction at the end of 5-day treatment is expressed as a percentage of starting total. (D) Striatal lesion formation. The percentage of animals with detectable post-mortem striatal lesions is shown. (E) Size of striatal lesions. Postmortem measurements of striatal lesions along the rostrocaudal axis. The asterisks indicate a P<0.05 *vs* wild-type according to One-way Anova and Tukey multiple comparison post-hoc test for panels A, B and E and to χ^2^ test for panels C and D.

Expression of hMTH1 in the transgenic animals protected against 3-NP-induced neurodegeneration. The first evidence of this protective effect was a significantly attenuated weight loss at day 5 of treatment in *hMTH1-Tg^+/+^* mice (Figure 5A). In wild-type animals, treatment with 3-NP causes a dramatic motor impairment as shown by their high (>3) mean neurological score (Figure 5B). Neurological score was defined as the highest score reached at any time of the observation period according to the following scale (modified from [31]): intermittent dystonia of one hindlimb: 1; intermittent dystonia of two hindlimbs: 2; permanent dystonia of hindimbs: 3; uncoordinated and wobbling gait or recumbency: 3; near death recumbency: 4. Transgene expression provided protection against this impairment and scores were progressively lower in hMTH1 hemizygous and hMTH1 homozygous mice (Figure 5B). hMTH1 activity was also associated with a striking decrease of mortality. While at 5 days 55% (11/20) of wild-type mice had died, the great majority of *hMTH1-Tg^+/−^* (13/16) or *hMTH1-Tg^+/+^* (13/16) remained alive (Figure 5C). Post-mortem examination of 3-NP-treated mice revealed detectable striatal lesions (namely macroscopically-evident pale striatal areas) in 77.7% of wild-type animals. These lesions were present in only 38.4% and 30% of *hMTH1-Tg^+/−^* and *hMTH1-Tg^+/+^* animals, respectively (Figure 5D). In animals showing detectable striatal lesion, a reduction in the mean lesion area was found in *hMTH1-Tg^+/+^* (3515±305 µ^2^, P<0.05 vs *wild type*) and *hMTH1-Tg^+/−^* (4152±511 µ^2^, NS) *vs* wild type mice (5262±528 µ^2^). Furthermore, the rostrocaudal extension of the lesions was significantly reduced in both *hMTH1-Tg^+/−^* and *hMTH1-Tg^+/+^* versus WT mice (Figure 5E). Thus, hMTH1 expression significantly protects the animals from the behavioural and neuropathological effects of 3-NP.

**Figure 6:**
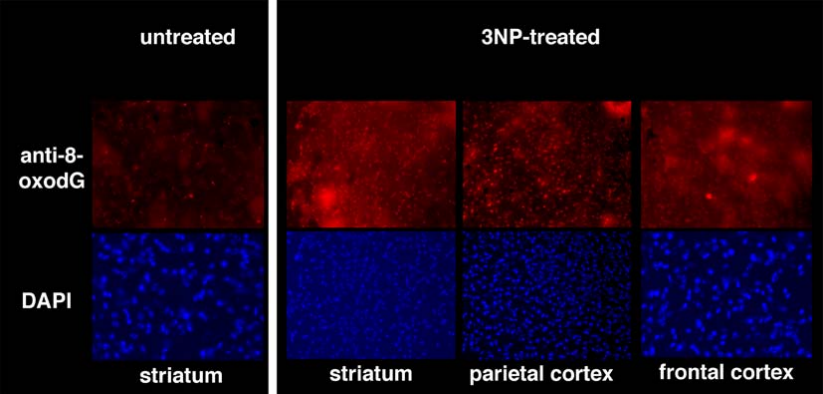
Oxidative DNA damage in the brain. Brains from wild-type mice that had been treated twice per day for 5 days with 60 mg/kg 3-NP were examined for DNA 8-oxodG by immunohistochemistry. Nuclei of striatum, parietal and frontal cortex were counterstained by DAPI.

While basal levels of DNA 8-oxodG in untreated animals were undetectable by immunohistochemical analysis of the oxidized purine, a 5-day exposure to 3-NP increased the level of this oxidized purine in the striatum and parietal and frontal cortex of wild-type mice (Figure 6). This indicates that this mitochondrial toxin produces extensive oxidative DNA damage.

**Figure 7:**
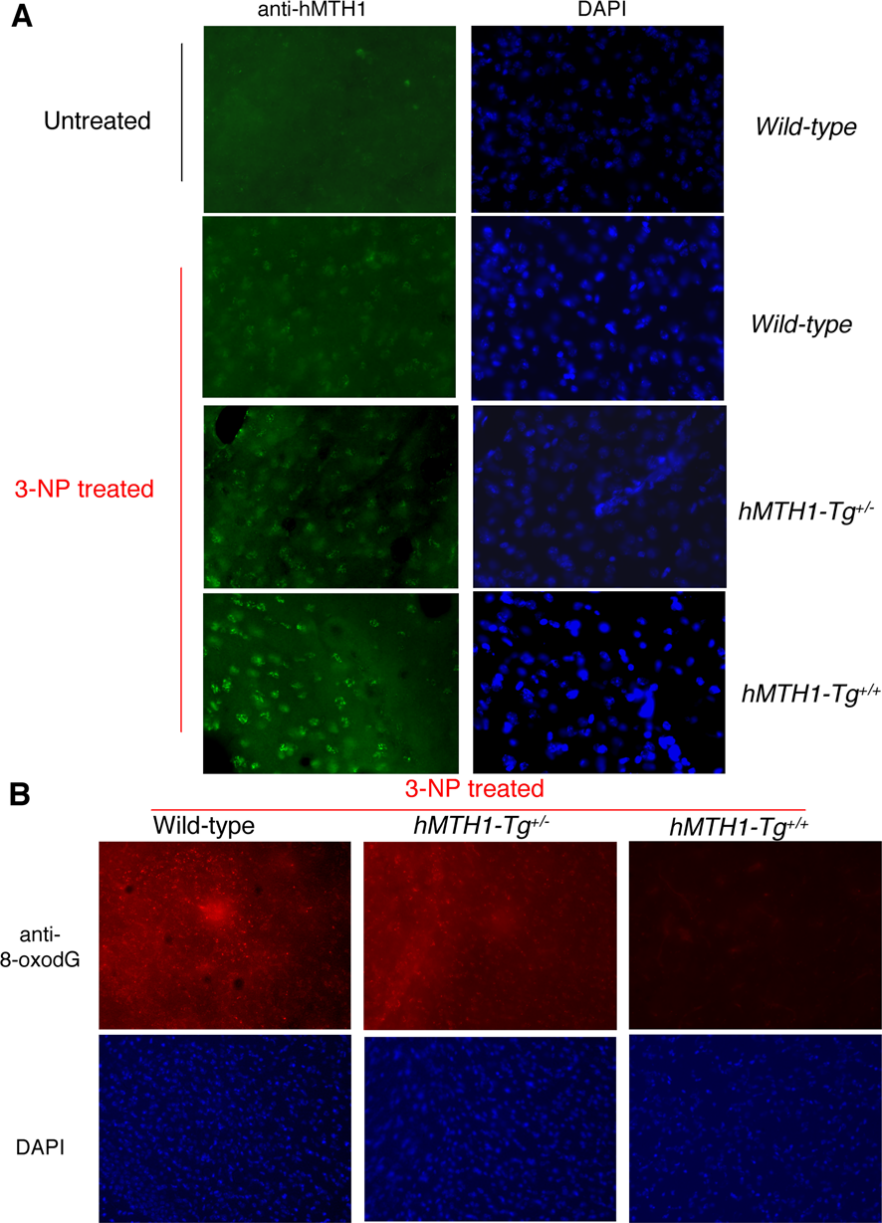
MTH1 expression and 3-NP-induced oxidative DNA damage in the brain. (A) Immunofluorescence of MTH1 in the striatum of untreated wild-type mice (top panel, left) or 3-NP-treated (60 mg/kg twice daily for 5 days) wild-type, *hMTH1-Tg^+/−^*, and *hMTH1-Tg^+/+^* animals. Nuclei of striatum counterstained by DAPI are shown in the right panels. (B) 8-oxodG immunoreactivity in the striatum of 3-NP-treated wild-type, *hMTH1-Tg^+/−^*, and *hMTH1-Tg^+/+^* animals. Nuclei of striatum counterstained by DAPI are shown in the bottom panels.

Although endogenous mMTH1 is normally undetectable, it can be visualized by immunofluorescence in the striatum of wild-type mice following 3-NP-treatment (Figure 7A). This suggests that the murine protein is induced in oxidatively stressed striatal cells. As expected, a progressively increasing signal for hMTH1 was observed in hemizygous *hMTH1-Tg^+/−^* and homozygous *hMTH1-Tg^+/+^* animals (Figure 7A). hMTH1 expression significantly reduced 8-oxodG levels in the major target area, the striatum, and increasing protection was observed in hemizygous *hMTH1-Tg^+/−^* and homozygous *hMTH1-Tg^+/+^* animals (Figure 7B).

These data establish an inverse correlation between the levels of DNA 8-oxodG and expression of the hMTH1 in the brain and suggest that, during the course of chemically induced neurodegeneration, a large fraction of this oxidative lesion derives from an oxidized dNTP pool.

It is possible therefore that the reduced levels of 8-oxodG pools afforded by the hMTH1 transgene, resulted in a diminished incorporation of 8-oxoG into DNA during repair of 3-NP induced oxidative DNA damage.

### Exogenous Expression of hMTH1 Protects Neural Progenitor Cells Expressing the *huntingtin* Gene against Cell Death

3-NP is a chemical model for HD-like striatal degeneration. We also investigated whether hMTH1 also conferred protection in a genetic model for HD. In this case the genetic alteration in the *htt* gene had already occurred and the contribution of oxidized precursors to the phenotype associated with an expanded CAG tract was studied. We used neuronal progenitor cell lines established from striatal primordia of wild-type or mutant *htt* knockin mice (*Hdh^Q7/Q7^* and *Hdh^Q111/Q111^*, respectively) in which the *htt* gene CAG repeat length is normal or expanded [22]. These nestin-positive cells have been immortalized with the tsA58 mutant of SV40 large T antigen and at the non-permissive temperature (39°C), similarly to the ST14A rat model [32], they cease proliferation and withdraw from the cell cycle.

**Figure 8:**
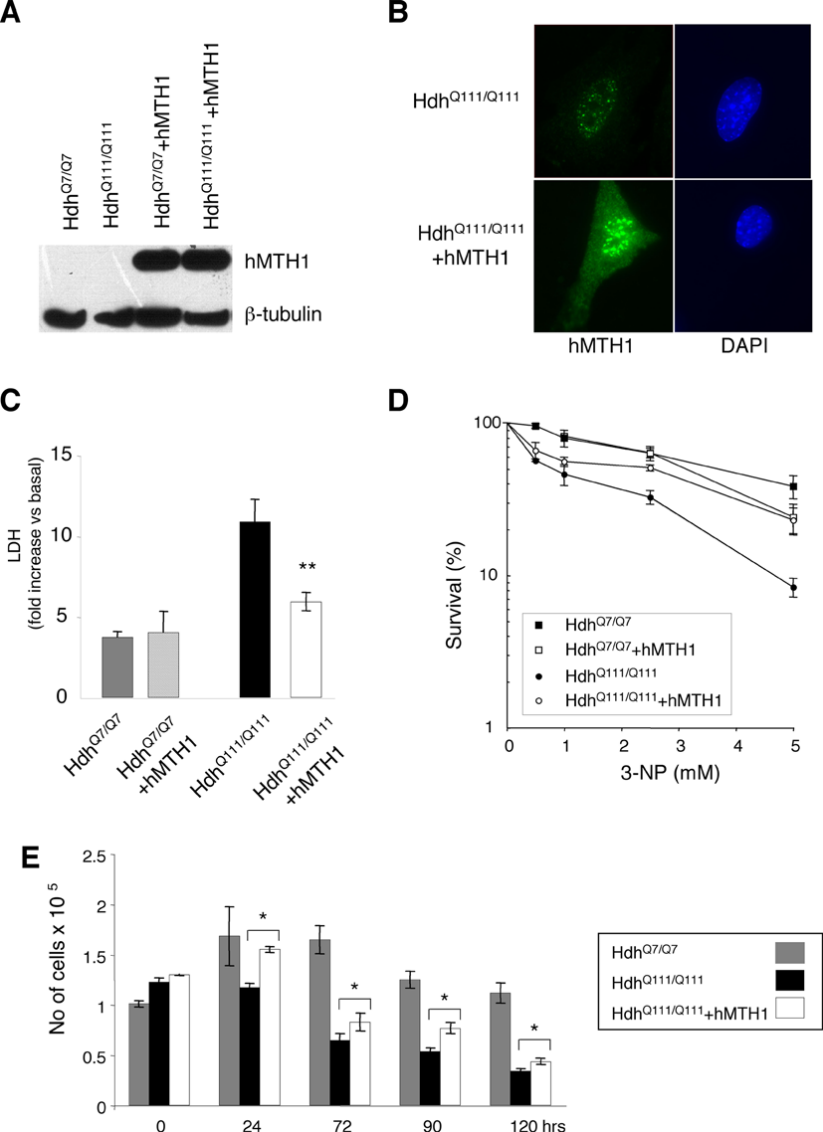
Sensitivity to 3-NP of striatal cells expressing hMTH1 and wild-type or mutant murine *htt*. Striatal cells derived from wild-type Hdh*^Q7/Q7^* and mutant *Hdh^Q111/Q111^* mice were transfected with hMTH1. (A) Proteins were separated and probed with an antibody against hMTH1. (B) Intracellular localization of hMTH1 (green fluorescence) in *Hdh^Q111/Q111^* and *Hdh^Q111/Q111^*+hMTH1. Nuclei were counterstained by DAPI. (C) LDH release. LDH release from striatal cells into culture medium was measured 24 hr after continuous exposure to 20 mM 3-NP. Hdh*^Q7/Q7^* (grey bar) and Hdh*^Q7/Q7^*+hMTH1 (dashed bar); *Hdh^Q111/Q111^* (black bars) and *Hdh^Q111/Q111^*+hMTH1 (open bar). Mean±SE, n = 4. (D) Clonal survival. Cloning efficiency was determined at 33°C after 24 hr continuous exposure to the indicated 3-NP concentrations. Mean±SD, n = 2. (E) Coulter counter assay. Survival of non-proliferating cells measured in a Coulter Counter. Mean±SD, n = 3. The asterisks indicate significant differences by Student's *t*-test (p values = 0.02) between *Hdh^Q111/Q111^* and *Hdh^Q111/Q111^*+hMTH1.

The vector expressing the *hMTH1* cDNA (pcDEBΔ) [23] was introduced into these cells by transfection and single clones with similar levels of hMTH1 expression were isolated (Figure 8A). A strong signal for hMTH1 was observed by immunofluorescence both in the nuclei and cytoplasm of the transfectants, while a weak hMTH1 signal in untransfected *htt* knockin cells showed mostly a nuclear localization. In Figure 8B is shown an example for a transfectant of the *Hdh^Q111/Q111^* cell line.

As previously reported [33], proliferating *Hdh^Q111/Q111^* striatal cells expressing mutant *htt* are more sensitive than *Hdh^Q7/Q7^* cells to killing by 3-NP as measured by LDH release (Figure 8C). Expression of hMTH1 protected *Hdh^Q111/Q111^* cells against 3-NP (p = 0.02; Anova test), but had no significant effect in cells expressing a wild-type *htt* gene (Figure 8C). Similar differences were revealed when survival was measured by clonal assays (Figure 8D).

To investigate whether hMTH1 was able to protect mammalian cells from toxicity associated with mutant *htt* expression even in the absence of DNA synthesis, cell cultures were shifted at the non-permissive temperature (39°C) to obtain a quiescent cell population. FACS analysis demonstrated that degradation of the mutant SV40 large T antigen led to a proliferative block in both *Hdh^Q7/Q7^* and *Hdh^Q111/Q111^* cells (data not shown). Expression of the mutated *htt* gene in *Hdh^Q111/Q111^* cells was associated with a dramatic time-dependent increase in cell killing, while a large percentage of normal *Hdh^Q7/Q7^* cells remained alive (Figure 8E). In these culture conditions expression of hMTH1 provided again a significant level of protection to *Hdh^Q111/Q111^* cells against cell death (Figure 8E). Growth at 33°C was unaffected by expression of hMTH1 in either *Hdh^Q7/Q7^* or *Hdh^Q111/Q111^* cells (data not shown).

From these results we conclude that hMTH1 is a major protective factor against cell death caused by a mutant *htt* gene both in proliferating and in quiescent cell culture conditions. These results also indicate that oxidized precursors modulated by hMTH1 plays a role in the phenotypic expression of striatal cell toxicity similar to that occurring in HD.

## Discussion

The accumulation of oxidative damage in brain DNA is a common feature of several neurodegenerative diseases [5]–[7], although evidence for a causal contribution of these DNA lesions to the disease process has been lacking. Our experiments with the transgenic mouse expressing the human 8-oxo-dGTPase hMTH1 indicate that oxidized dNTPs are important contributors to basal levels of DNA oxidation in vivo. In addition sanitization of the oxidized pool by hMTH1 significantly lowers DNA 8-oxoG levels in several tissues of mice exposed to the in vivo oxidant paraquat. This broad-spectrum pesticide exerts its toxic effects in several organs including lung, heart, liver, kidney and brain [28]. Although the mechanism of its toxicity is not fully elucidated, paraquat has been shown to induce toxicity by stimulating oxygen utilization via redox cycling and generating reactive oxygen intermediates [34]. Thus, detection of increased DNA 8-oxodG in paraquat-treated rats identified brain, lung, heart, liver and kidney as target organs of paraquat-induced oxidative DNA damage [30]. We also observed accumulation of DNA 8-oxodG in several organs of wild-type mice following repeated exposures to paraquat. It is striking that protection provided by hMTH1 overexpression against paraquat-induced DNA damage occurred in the same organs (brain, heart and small intestine) protected against endogenous oxidation. This indicates that oxidized dNTPs in these organs are a particularly important target for both exogenous and endogenous sources of DNA damage. Thus, hMTH1 represents a general mechanism of defence against accumulation of oxidized purines in nucleic acids produced either endogenously or by an exogenous oxidative stress.

Our in vitro observations using MEFs derived from hMTH1-Tg mice support this conclusion and indicate that expression of hMTH1 leads also to protection against cell death induced by an in vitro exposure to an oxidant.

Experiments with knockout *Mth1^−/−^* mice established a connection between mMTH1 activity and the levels of DNA and RNA 8-oxodG in dopaminergic neurons following exposure to a selective neurotoxin in a PD model [19] and in hippocampal microglia during kainate-induced excitotoxicity [35]. In those animals, abrogation of mMTH1 expression had no measurable impact on the disease, however.

In contrast, transgenic hMTH1 expression revealed important connections between nucleotide pool oxidation, HD-like neurological degeneration in a targeted area of the brain, and neurological symptoms. Neurological symptoms that resemble HD were produced in vivo by treating animals with 3-NP, an inhibitor of complex II of the mitochondrial respiratory chain. hMTH1 expression protects against HD-like neurodegeneration in vivo and this is associated with decreased levels of DNA 8-oxodG in the striatum. The dramatic attenuation of HD symptoms in transgenic animals was reflected in a significantly reduced size of the chemically–induced striatal lesions as well as in an increased survival. Since hMTH1 protects against the accumulation of both 8-oxodG and 2-hydroxyadenine in nucleic acids [16], our findings suggest that oxidized purine nucleotide precursors of DNA and RNA might be causal factors in HD-like neurodegeneration.

Striatal neurodegeneration in 3-NP experimental model of HD most likely occurs in terminally differentiated, non-dividing neurons. Thus any impact of hMTH1 on nuclear DNA replication is unlikely to be significant. Mitochondrial DNA stability is a plausible alternative since impaired mitochondrial respiration and ATP production play a central role in HD [36]. High levels of hMTH1 in MEFs derived from the transgenic mice are localized in the mitochondria, in agreement with previous reports in human cells [23]. Intense staining for 8-oxodG in several areas of the brain of wild type mice was produced by the 5-days exposure to 3-NP. It is interesting that in a mouse model for PD induced by systemic administration of 1-methyl-4-phenyl-1,2,3,6-tetrahydropyridine, *MTH1*-null mice accumulated higher levels of 8-oxodG in mitochondrial DNA of the striatum than wild-type mice and this triggered neuronal dysfunction [19]. It is possible that a large fraction of oxidative damage induced by 3-NP and identified by immunostaining also resides in mitochondrial DNA. We suggest that the protective role played by hMTH1 in this experimental model of HD is, in all probability, a defensive mechanism against mitochondrial degeneration induced by the neurotoxin.

The accumulation of 8-oxodG in neurodegerative diseases such as PD, AD or ALS is paradoxically accompanied by up-regulation of repair enzymes involved in the control of oxidative DNA damage. Thus, increased levels of hMTH1 [37], hMYH [38], or the mitochondrial form of hOGG1 [39] have been reported in the mitochondria of neurons from *substantia nigra* of PD patients. This up-regulation of several DNA repair enzymes has been interpreted as a general marker of oxidative stress associated with this disease. We observed increased immunostaining for mMTH1 in the affected areas of the brain of wild-type mice induced by 3-NP to show HD-like neurodegeneration. This suggests that in this experimental model of HD, similarly to other neurodegenerative diseases, increased levels of 8-oxodG are accompanied by an up-regulation of MTH1 expression.

hMTH1 also protected neurons in a genetic model of HD, in which progenitor striatal cells express an expanded CAG region of the mouse *htt* gene. The role of oxidized purines in HD is probably multifaceted and OGG1 can contribute to expansion of triplets in the *htt* gene. Thus ablation of *Ogg1* in R6/1 mice, a model of HD which harbours a transgene containing exon 1 of the human *HD* gene with an expanded CAG repeat, impairs age-dependent triplet expansion [40]. In the model cell lines we used, CAG expansion in the *htt* gene has already occurred, however. We show here that low levels of 8-oxoG in nucleic acids provided by hMTH1 overexpression leads to protection against mutant *htt*-associated toxicity. Thus proliferating progenitor striatal cells are protected against selective vulnerability induced by exposure to the 3-NP mitochondrial toxin [33]. It has been shown that this is due to a non-apoptotic form of cell death caused by mitochondrial membrane depolarization [33]. In addition, mutant htt is also toxic to quiescent cultures without exogenous stress, possibly because of activation of specific apoptotic pathways [33]. hMTH1 expression provided a safeguard effect in both settings suggesting that oxidized triphosphates play a major role in both killing mechanisms. This indicates that the oxidative DNA damage modulated by hMTH1 can be not only causative for HD-like disease (3-NP model) but may affect some phenotypic manifestations of this neurodegenerative disease. To address the possible pathogenetic role of oxidative DNA damage in HD, a more stringent experimental approach (e.g. cross breeding hMTH1 overexpressing mice with HD mice) is also planned. This will be informative on the possible role of DNA 8-oxodG levels in controlling the degree of triplet expansions as well as on the progression of the disease.

Finally, the efficient hMTH1-mediated elimination of oxidized RNA precursors we show here might be particularly important in protecting vulnerable neuronal populations against translational errors following mRNA oxidation [41]. This second function of hMTH1 in preventing transcriptional errors [42],[43] might play a minor role in the dramatic neurotoxicity associated with an acute exposure to 3-NP-induced oxidative stress. In human HD, however, in which neurodegeneration requires a long period of time to occur and neuronal populations are probably exposed to a less dramatic level of oxidative insults, hMTH1 might become a major safeguarding mechanism.

## Materials and Methods

### Construction of Transgenic Mice and Analysis of Mendelian Ratio

The construct used to build the transgenic mice was obtained using a BamH1-EcoRV fragment (509 bp) derived from pcDEBΔ [23] encoding the *hMTH1* cDNA and subcloned into the gWIZ vector (Gene Therapy systems) under the control of the CMV promoter. The MscI-KpnI fragment (2481 bp) was purified and used for pronuclear injection of (C57/Bl6×DBA2) F2 zygotes using standard procedures. Injected embryos were implanted into pseudopregnant (C57/Bl6×DBA2) F1 foster mothers to complete their development. All mice were genotyped by PCR using DNA isolated from tail tips following standard proteinase K digestion and phenol/chloroform extraction. The forward primer was located in the vector (5′-TCTTTTCTGCAGTCACCGT-3′) and the reverse primer in the *hMTH1* cDNA (5′-GGTCTCTCCTTCTTGCAC-3′). The 200 bp amplification product was obtained by 1 cycle at 95°C for 5 min, 30 cycles of 95°C for 1 min, 57°C for 2 min and 72°C for 2 min, followed by 1 cycle of 72°C for 10 min. To assess the transgene copy number, 9 µg of MscI-KpnI DNA fragment was spiked with various amounts equivalent to 1 (4.1 pg), 2, 5, 10, 20, 40 copies. DNA (10 µg) from tail tips was digested with BamHI overnight at 37°C and applied to electrophoresis on 0.8% agarose gel and transferred onto Hybond N^+^ nylon membrane (Amersham) by a standard alkali transfer method. The filter was hybridized with the MscI-KpnI fragment of *hMTH1* cDNA labeled with [α-^32^P]dCTP using a labeling kit according to manufacturer's instructions (Random primed DNA labeling kit; Roche). Quantification was performed using a phosphorImager (Canberra Pakard).

Five couple of hemizygous mice for hMTH1 were crossed to analyse the Mendelian segregation and the offspring analysed by FISH for the presence of transgene.

### Fish Analysis

Further confirmations of the genotypic constitution of transgenic mice were obtained by Fluorescent *In Situ* Hybridisation (FISH) analysis. Fixed metaphases of bone marrow cells, extracted from femurs of mice intraperitoneally injected 2 hrs before sacrifice with 4 mg/kg b.w. of Colchicine, were denaturated 2 min at 72°C in 70% formamide and then hybridised overnight with 200 ng/µl of *hMTH1* digoxigenated probe preparated by DIG-Nick translation mix (Roche) according to manufacturer's instructions. After washing with 50% formamide at 37°C the probe was detected by sheep anti-digoxigenin-rhodamine antibodies and the chromosomes were counterstained by DAPI. Ten complete (40 chromosomes) metaphases were analysed for each mouse.

### Expression of hMTH1

RNA was isolated from indicated organs using the RNeasy Mini kit following the manifacturer's instruction (Quiagen). The RNA pellet was briefly air-dried, resuspended in RNase-free water and stored at −80°C. The RNA was retro-transcribed into cDNA by using the SuperScript One-step RT-PCR with platinum Taq (Invitrogen). Total RNA (500 ng) was used for the amplification following the manufacturer's instructions. In order to evaluate *hMTH1* expression, primers were designed to specifically allow the detection of the human transcript without pairing with the mouse one. The forward and reverse primers were respectively 5′-AGGAGAGCGGTCTGACA-3′ and 5′- GGCCACATGTCCTTGAAG-3′. PCR conditions in the amplification were 55°C for 30 min and 94°C for 2 min, 35 cycles of 94°C for 1 min, 54°C for 2 min, 72°C for 2 min. The final extension step was 10 min at 72°C. PCR products were analysed on a 2% agarose gel. We used as internal control the amplification of the mouse Gapdh (forward 5′-ATCCACTGGTGCTGCCAA-3′ and reverse 5′-CCACCCTGTTGCTGTAG-3′).

### Western Blot Analysis of hMTH1 in Organs and Cell Extracts

Animals were killed by cervical dislocation and organs were collected on ice. To prepare cell extracts 30 mg of each organ was minced in USA buffer (1 ml) [Tris-Hcl 50 mM (pH 7.4), NaCl 250 mM, EDTA 5 mM (pH 8), 0.1% Triton, NaF 50 mM, NaOVa 0.1 mM and a protease inhibitor cocktail tablets (Complete mini, Roche) by using a Mixer Mill 300 homogenizer. To prepare cell extracts from MEFs, 10^6^ cells were lysed in Ripa Buffer [Tris-Hcl 50 mM (pH 7.4), NaCl 150 mM, EDTA 1 mM (pH 8), 1% NP40, NaF 1 mM, and a protease inhibitor cocktail tablets (Complete mini, Roche)] for 1 hr on ice and then centrifuged at 14,000 rpm for 30 min. Cytosol and mitochondrial cell extracts were prepared using the Mitochondria isolation kit (Pierce) according to manufacturer's instructions. Mitochondria were lysed in Ripa Buffer to obtain mitochondrial protein fraction. Protein concentration was evaluated using the Bradford method and 20–40 µg of total extract was separated on 12%- (for whole cell extracts) and 18%- (for mitochondrial and cytosolic fractions) -SDS polyacrylamide gels, transferred to nitrocellulose membranes (Whatmann) with a TransBlot cell apparatus (Bio-Rad), and probed overnight at 4°C with anti-hMTH1 antibody [23] followed by the appropriate secondary antibody. ECL detection reagents (Invitrogen) were used to develop the blots. Loading controls for whole cell extract were β-tubulin (1∶5000) and cytochrome *c* (1∶500) for mitochondrial fractions.

### Preparation of Mouse Embryo Fibroblasts and Cell Cultures

MEFs were prepared from embryos derived from wild type or *hMTH1-Tg^+/+^* pregnant females sacrificed after 13 days of gestation. After forceps dissection from the uterus and placenta embryos were rinsed in PBS, minced with scissors and the tissue suspension was incubated in a solution of 0.25% (w/v) trypsin/ 1 mM EDTA at 37° for 10 to 15 min with vigorous stirring. Then Dulbecco's modified Eagle's medium (DMEM) supplemented with 10% fetal bovine serum, penicillin (100 U/ml), and streptomycin (100 µg/ml) (complete medium) was added and large clumps were allowed to settle for 2 min. The decanted cells were centrifuged at 200 g for 5 min at room temperature and resuspended in complete medium. MEFs spontaneously immortalised upon in vitro passaging and were grown routinely in complete medium at 37°C in a 5% CO_2_ atmosphere (90% nominal humidity).

### MTT Assays

Four thousand cells/well were plated in 96-well plates, treated the next day with various concentration of KBrO_3_ (5–40 mM; Sigma) in PBS/Hepes (20 mM) for 30 minutes at 37°C, washed in PBS 1× and incubated in DMEM for 48 hrs. The 3-(4,5-dimethylthiazol-2-yl)-2,5-diphenyltetrazolium bromide) (MTT) (Sigma) solution (20 µl, 5 mg/ml in PBS) was then added and plates were incubated for 3 hr at 37°C. Following addition of 1 volume of DMSO, plates were incubated for 30 min in the dark. The solubilized formazan was quantified at 570 nm, using a microplate reader (DINEX, UK).

### Analysis of 8-oxodG and 8-oxoG

Nucleic acids were isolated as previously described [25] with minor modifications. Briefly, cells were trypsinized and cell pellets lysed in 3 M guanidinium thiocyanate/10 mM deferoxamine mesylate (DFOM). Cell lysates were transferred to Phase Lock gel tubes, mixed with an equal volume of Phenol-Chloroform-Isoamyl alcohol pH 6.7 and centrifuged. The acqueous phase containing both DNA and RNA was extracted with one volume of Chloroform-Isoamyl alcohol and precipitated with cold isopropanol at −80°C. Nucleic acids were centrifuged, washed with cold 70% ethanol and air dried. For 8-oxodG and 8-oxoG determinations pellets were dissolved in 30 µM DFOM solutions and digested with 2–4 units Nuclease P1 and 10 units alkaline phosphatase at 50°C for 1 hr. Samples were prepurified through micropure EZ filters and transferred to HPLC autosampler vials. Separation of digested DNA components was accomplished with a Beckman System Gold HPLC apparate equipped with diode array UV detection. Normal nucleosides were detected at 254 and 290 nm. Electrochemical detection of 8-oxoG and 8-oxo-dG was performed with an ESA Coulochem II detector. Guard, conditioning and 5011 high sensitive analytical cell were in line with graphite filter elements. The whole system featured PEEK tubings. Solvent system consisted of 5% MeOH and 95% 50 mM potassium phosphate. Flow rate was 0.5 ml/min. The LC-18-DB (Supelco, 75×4.6 mm) analytical column was equipped with a YMC ODS-AM 12 nm 5 µm guard column. The Beckman's Karat analytical software was used for data analysis.

### In Vivo Treatments with Paraquat

Animals were kept under standardized temperature, humidity, and lighting conditions with free access to water and food. Animal care and use followed the directives of the Council of the EC (86/609/EEC). Two groups (wild-type and hMTH1-Tg) of 8-weeks old animals were treated with 10 mg/kg paraquat CL tetrahydrate. Animals of both sexes (1∶1 ratio) were included in each group. Mice were intraperitoneally injected with either saline or Paraquat at 2-days intervals for a total of 5 doses. Animals were killed by cervical dislocation 2 days after the end of treatment and excised organs were washed with ice-cold PBS. Washed tissues were snap-frozen in liquid nitrogen and thawed tissues were finely minced in lysis buffer [10 mM Tris HCl (pH 8.0), 10 mM EDTA, 10 mM NaCl, and 0.5%SDS]. DNA was extracted by a high-salt protein precipitation method. Briefly, following lysis tissues were digested with RNase at 37°C for 1 hr and protease (Qiagen) at 37°C overnight. Proteins were precipitated by adding NaCl to 1.5 M, and DNA in the supernatant was collected by addition of 2 volume of ethanol. 8-oxodG was measured by HPLC with electrochemical detection HPLC/EC as described previously [24].

### In Vivo Treatment with 3-NP

3-NP (Sigma-Aldrich) was dissolved in PBS (pH adjusted with NaOH) and administered twice a day (h 9.00 and 16.00) for 5 days at the dose of 60 mg/kg i.p. Wild-type (20), hMTH1-Tg^+/−^ (16) and hMTH1-Tg^+/+^ (16) mice were used. Animals of both sexes (1∶1 ratio) were included in each group. Baseline body weight was determined before the start of the experiment. Weight changes were monitored daily, immediately before the 9.00 injection. The occurrence of dystonia and/or gait abnormalities was evaluated twice a day. For each animal, the highest score reached at any time of the observation period was considered, according to the following scale: intermittent dystonia of one hindlimb: 1; intermittent dystonia of two hindlimbs: 2; permanent dystonia of hindimbs: 3; uncoordinated and wobbling gait or recumbency: 3; near death recumbency: 4 (modified from ref. [31]). Two hours after the last injection, surviving animals were killed by decapitation; brains were removed and immediately frozen. For each brain, serial 20-µm coronal sections were cut on a cryostat microtome, stained with Cresyl violet and examined by light microscopy. In animals showing a striatal lesion, the rostrocaudal extension of the lesion and lesion areas were measured in mm and µ^2^, respectively.

### Immunofluorescence

Untreated and 3-NP-treated animals were killed by decapitation and the brains were removed immediately and cryopreserved in isopentane chilled on dry ice. Tissue was cut in 20 µM thin section, these were placed on Superfrost/Plus microscope glass slides and used for localization of 8-oxodG (anti-8-oxo-dG; Trevigen) and hMTH1. To reveal 8-oxodG, slides prepared were dried overnight at room temperature and fixed for 10 min in 70% ethanol at −20°C then washed in PBS 1× and incubated in a RNase solution (10 mM Tris-HCl (pH 7.5) 1 mM EDTA, 0.4 mM NaCl, 100 µg/mlRNase) for 1 hr at 37°C. To detect the 8-oxodG in nuclear DNA slides were further denatured in 4NHCl for 7 minutes, blocked in 10% fetal bovine serum for 1 hr at 37°C and incubated overnight at 4°C with anti-8-oxodG monoclonal antibody [1∶300 in 10 mM Tris-HCl (pH 7.5) 10% serum]. On day 2 the slides were rinsed 2×10 min in PBS 1× containing 0.025% triton X-100 and incubated with a TRITC secondary antibody (goat anti-mouse) in serum 3% for 1 hr a 37°C and nuclear staining was obtained with 200 µg/ml DAPI. To reveal hMTH1, slides were dried overnight at room temperature, fixed in absolute methanol at −20°C for 15 min and in ice-acetone for 30 sec, then dried and frozen. Slides were then rinsed 3 times for 10 min each in PBS 1× containing 0.025% triton X-100 and incubated overnight at 4°C in a humidified chamber with the primary human anti-MTH1 polyclonal antibody diluited 1∶200 in 3% serum. Slides were then rinsed 3 times for 10 min in PBS 1× containing 0.025% triton X-100 and incubated with a secondary FITC antibody (goat anti-rabbit 488) in serum 3% for 1 hr a 37°C and nuclear staining was obtained with 200 µg/ml DAPI. Slides were mounted with Antifade and analyzed using a Leica DMRB fluorescence microscope equipped with a CCD camera. The images were processed using the IAS 2000 Delta System software.

### Striatal Cell Cultures, DNA Transfection, and Measurements of Cell Death

Cells derived from wild-type and mutant *htt* knockin mice (*Hdh^Q7/Q7^* and *Hdh^Q111/Q111^*) (a kind gift of Elena Cattaneo, University of Milan) were routinely grown at 33°C in high-glucose DMEM supplemented with 10% fetal bovine serum, penicillin (100 U/ml), and streptomycin (100 µg/ml) (complete medium). Exponentially growing *Hdh^Q7/Q7^* and *Hdh^Q111/Q111^* cells were transfected (Lipofectamine) with pcDEBΔ and stable Hygromycin (200–300 µg/ml) resistant clones were isolated after approximately 20 days in selective media. Survival after treatment with 3-NP was determined by lactate dehydrogenase (LDH) release and clonogenic assay. For LDH release, cells were plated in 48 well plates in complete medium and incubated at 33°C. Serum-free DMEM was added 24 hr later and cultures were exposed to 3-NP for the next 24 hr. LDH release into the medium was evaluated using a citotoxicity detection kit (Roche). Briefly,100 µl of medium was mixed with 100 µl of the LDH substrate mixture in a 96 well plate. After a 30 min incubation at room temperature the absorbance was measured using a spectrophotometer. Results are expressed as fold increase *versus* control and represent means±standard error of at least 4 independent experiments, assayed in triplicates. Statistical analysis was carried out by the unpaired t-test and a p value<0.05 was considered significant. For clonal assays, 100–200 cells were treated with the drug 18 hr after seeding, cultures were washed 24 hr later, fed with complete medium and 1–2 weeks later surviving colonies were fixed, stained with Giemsa and counted.

To obtain a non-proliferating cell population, cultures were first incubated at 33°C for 8 hr in complete medium. Before transferring the cultures at 39°C complete medium was replaced by serum-deprived medium (F-12/DMEM, 5 mg/l insulin, 100 mg/l transferrin, 20 nM progesterone, 30 nM selenite, 60 µM putrescine, 2 mM glutamine. 0.11 g/l sodium pyruvate, 3.7 g/l sodium bicarbonate and 3.9 g/l HEPES)[30]. To measure survival, cells were trypsinized, resuspended in PBS and counted using a Coulter Counter machine (ZM, Coulter Instruments).
